# Providing Adaptation Solutions to the Problems Faced by Adoptive Families

**DOI:** 10.7759/cureus.53262

**Published:** 2024-01-30

**Authors:** Nafiseh Heshmati Molaie, Roya Koochak Entezar, Fatemeh Golshani

**Affiliations:** 1 Department of Psychology and Educational Sciences, Central Tehran Branch, Islamic Azad University, Tehran, IRN

**Keywords:** adoption, orphan, orphan children, adoptive families, adaptation solutions, adoption problems, psychiatry and psychology

## Abstract

Background: Adoption is frequently viewed as a way to complete the family because of the infertility that some families experience and the desire for kids and teenagers, especially orphans, to have a loving family.

Aims: This work intends to identify and propose adaptation solutions to address the psychological problems faced by adoptive families. By doing so, it is hoped that the mental health and overall well-being of individuals and society as a whole will be enhanced.

Materials and methods: In the first stage of this study, the grounded theory approach based on Strauss and Corbin’s methodology was used to interview representatives of adoptive families in Iran in 2022 and identify the problems they experience. In the second stage, the same approach and methodology were used to interview representatives of experts. In the second stage, the results of the first stage were presented to a sample of purposefully selected experts, who subsequently proposed solutions for the families to adapt to their problems. Data analysis was conducted using MAXQDA 2020 software (VERBI Software, Berlin, Germany). Ethical requirements were followed at every stage of the study.

Results: Four problem categories were identified in the first step of the study: legal-psychological problems, problems related to the growth of the child in an environment of social harm, attitude and worldview, and the resilience of the adoptive couple. In the second step of the study, four groups of solutions for psychological adjustment were extracted: explaining the issue of what, how, and why of adoption (correct identification of the problems faced by adoptive families, clarification of concepts and demystification, the need for research to address other gaps in the knowledge infrastructure, and the importance of critical thinking education), operational solutions (supervising the adoption database to become more efficient, unitization of adoption national institutes in each province, selection of officials based on both factors of professional and complete mental health, alignment of the provisions and other related legal matters between the involved institutions, and utilization of media influence are recommended, interactional solutions for family resilience (cognitive-therapeutic interactions for mental health based on flexibility, raising the family's social capacity through active counseling, improving problem-solving skills, fostering family self-efficacy, creating purposefulness, and appropriate beliefs to predict a bright future), attitude-changing solutions (redefining social norms and facilitating the adoption of desired attitudes by employing social psychology principles, exploiting the power of media and influential figures, employing techniques derived from the psychology of learning, establishing conducive conditions within the framework of individuals' cognitive dissonance to modify their attitudes, and employing persuasion strategies).

Conclusion: Legislators and law enforcers, adoptive families, psychologists and consultants working in this field, and physicians specializing in infertility treatment can benefit from the findings of this study.

## Introduction

Infertility is one of the most significant problems for the majority of couples. Recent attention on the relationship between infertility treatments, the use of assisted reproductive techniques, and the incidence of mental health disorders has gained much attention. There is a reported increase in the incidence of depression in women who use assisted reproductive techniques [1].

Social work plays a key role in assisting families and orphans. Understanding the challenges of social work will enable the teams to come up with effective solutions [2].

Every year, about 2,000 children from various regions of Iran are placed with adoptive families due to their circumstances. With the approval of judicial authorities, qualified families and individuals are granted guardianship for children who do not have validated guardianship documentation. A set of priorities has been established for the custody of these children. There are over 10,000 children in the country who lack effective guardians. Moreover, there are about 11 families seeking adoption for each child in Iran [3].

Socioeconomics, diverse cultures, ethnicities, and traumatic histories can impact the adoption process. Using the competencies to ground the work, child life specialists focus on the therapeutic relationship, attachment, and bonding while also understanding the grief and loss experienced by the biological parents [4].

While some research, such as Brodzinsky and Schechter's empirical work, has shown that adopted children are more vulnerable to a host of psychological and school-related problems compared to their non-adopted peers and that the rate of referral of adopted children to mental health facilities is far above what would be expected given their representation in the general population, our understanding of the basis for these problems remains unclear [5].

Adoptive parents, according to Goldberg's research, had significant worries about their children's psychosocial challenges when compared to their academic achievements. Across the sample, parents were more concerned about the children's socio-emotional growth and independence than their academic progress [6].

Choosing an appropriate adoptive family and preparing the child to go to a new family is important, but that is not enough. Because, according to some research studies, such as Rushton’s review, problems of families after adoption that may cause unsuccessful adoption and return of the child to the previous environment should be considered. The article recommends that adoption research needs to be considered as an integral part of general research into placement choices for children. Looking to the future, the commissioning of large-scale studies is recommended to gain a lifelong perspective on adoption, identify predictors of outcome, the consequences of contact arrangements for all parties, and determine the cost-effectiveness of different types of adoption support [7].

Adoptive parents can facilitate psychological connections between children and their biological families if they receive psychological assistance and are encouraged not to delay discussing adoption issues with their children. In other words, they can maintain the connection between the past and the present and accept the past as a part of the children's lives [8]. In another example regarding the significance of psychological support for parents, and with reference to the findings of one research study [9], it has been observed that adoptive couples often encounter concerns expressed by significant others in their lives, leading to the experience of negative emotions. Prejudice among the people in the vicinity, insufficient family support, and apprehensions regarding the adopted child's past have been identified as key contributors to the parents' concerns.

Additionally, one study [10] found that empowering parents regarding their children and associated issues can yield positive outcomes.

The current research aims to identify the problems experienced by adoptive families and propose solutions to help them adapt to these issues. The data were collected in September 2022 from various cities in Iran. In addition, interviews were conducted with experts to collect solutions for families to adapt to their problems. The findings of this grounded theory research were subsequently presented as a model.

## Materials and methods

The use of qualitative methods in scientific inquiry is on the rise [11]. We used grounded theory, a widely recognized and popular research method because it builds on the actual context of phenomena and allows the theory to emerge organically from the data that is collected through robust, accurate, and impartial procedures [12].

The research was conducted in two stages, with the grounded theory approach being employed in both stages. In the first stage, an interview was conducted with a representative from each adoptive family. All the families were among the volunteers who had become familiar with this research after viewing the invitation to participate in the study posted on the Internet. The criteria for inclusion in the study's sample were the expiration of the six-month trial period and the issuance of a permanent guardianship decision by an Iranian court. The problems of adoptive families were identified during this stage. In the second stage of the study, the results of the first stage were presented to experts, and through interviews with them and data analysis, adaptation solutions to the problems faced by adoptive families were determined.

The interview transcripts were initially coded with Strauss and Corbin's systematic approach in mind [13]. Before the meaningful units are coded and labeled in the initial coding stage, each of the observations collected based on purposeful and information-oriented sampling must be examined multiple times, and the important segments that contain information must be separated from those that do not [14]. The same measure was taken in the current study. The initial code is a word or phrase brimming with the concepts of a meaningful unit and can be in various formats [15]. The data in the present study consisted of transcriptions of interviews. Therefore, the text of the interviews was initially coded, and the participants confirmed that the codes were appropriate. According to [16], additional writings on the primary codes led the researcher to identify categories. Then, the codes that were meaningfully related to one another were grouped or classified [17]. Put another way, following [16], the researchers started with the open coding and axial coding processes and were directed toward the categories.

In this research, MAXQDA software 2020 (VERBI Software, Berlin, Germany) was used to record data and conduct qualitative research. This software program is considered the newest and most up-to-date qualitative data analysis tool [18]. In the first phase of the research, a representative from each adoptive family (selected at their discretion) was interviewed. The interviews were recorded and transcribed verbatim. The findings derived from the data analysis were then presented in the research, ensuring that the privacy of the participants was upheld.

Theoretical saturation is assumed to have been reached when all information sources provide similar information to the researcher and no new category emerges. However, it has been suggested that the researcher continue with a few additional interviews to confirm this perception [18]. Therefore, although the interviews reached theoretical saturation after the eighth interview, they continued until the 13th interview. Ethical requirements were taken into account at every stage of the research.

First, the participants were given a thorough explanation of the procedure and purpose of the study, and they voluntarily agreed to participate in the research. The privacy of individuals was respected. The interview with the research participants in the first stage was conducted in a manner that ensured minimal impact on them. They were instructed to simply narrate their adoption story in their own words and highlight any challenges they faced after the adoption. In the second phase, after experts were chosen using purposive sampling, they were treated with courtesy, the utmost respect for their social dignity, and appreciation. The research ethics committee of Islamic Azad University, Central Tehran Branch, Tehran, Iran issued approval (approval number: IR.IAU.CTB.REC.1402.090).

## Results

In the first phase of the study, the sample consisted of 84.6% female participants and 15.38% male participants. Every individual possessed either a diploma or a higher level of education. They were in the age range of 30 to 55 years and the middle range of socio-economic level. A significant proportion of families (69.2%) experienced infertility issues, while the remaining families (30.8%) did not encounter such problems and opted for adoption as their primary choice. The sample population consisted of 15.3% single mothers and 84.6% couples. To ensure sample diversity, participants were recruited from different provinces in Iran.

The problems that adoptive families experienced were identified by extracting codes from interviews conducted with representatives of each adoptive family. The conceptual relationships between these codes were then recognized, and they were categorized accordingly. After expert consultations were applied and the codes were modified, a total of 353 initial codes were obtained after the coding process.

Following the open coding and axial coding processes, we proceeded with selective coding. Four key concepts emerged during the first phase of the research: child development in the context of social harm, legal-psychological problems, attitude and worldview issues, and the problems associated with the couples' resilience. The issues pertaining to attitude and worldview appeared to be casual conditions.

In addition, the families were asked after the interviews if they were satisfied with adopting a child, and all thirteen families responded positively. When asked about the difficulty they sensed in their duties compared to those of biological parents, 10 out of 13 responded by stating that they did not believe their challenges were greater; "the only distinction lies in the type of certain problems we encounter." For example, biological children may ask their parents, "Why did you give birth to us?", while an adopted child may ask his adoptive parents, "Why did you adopt me?". The answer to each of these two questions has its challenge but is different from the other.

In the study's second phase, the findings from the first phase were shared with experts who specialized in the relevant categories and concepts. Subsequently, interviews were conducted with these experts, and the interviews were recorded with their consent. By the end of the same day, the interviews were transcribed verbatim and coded using MAXQDA 2020 software, following the same methodology as the first phase of the research. A total of 149 codes were acquired. The researchers utilized their understanding of codes and guide notes to facilitate a systematic coding process, progressing from categories to concepts.

Four concepts were extracted from the second stage of research: explain the what, how, and why of adoption; operational solutions; interactional solutions for family resilience; and attitude-changing solutions.

The first concept was to explain the what, how, and why of adoption. It consisted of four categories: correct identification of the problems faced by adoptive families; clarification of concepts and demystification; the need for research to address other gaps in the knowledge infrastructure; and the importance of critical thinking education. The first category, correct identification of the problems faced by adoptive families, emerged from the codes: knowledge of the philosophical, theological, and mythological foundations of adoption and correct diagnosis of the problem. The second category, clarification of concepts and demystification, emerged from the codes: popularization of science and demystification, disillusionment of cultural concepts, and creation of a suitable context for familiarization with people with different perspectives. The third category, the need for research to address other gaps in the knowledge infrastructure, emerged from the codes: conducting comparative research after considering relevant notions, the necessity of classifying cultural attitudes, the pathology of child adoption in different dimensions, and the examination of psychological development. Lastly, the fourth category, the importance of critical thinking education, was derived from the following codes: the practice of thinking about how one person's situation affects the entire society, focusing on universal values, and inducing questions in people's minds that will steer them away from dogmatic thinking.

The second concept was operational solutions. These solutions can be categorized into five main areas: supervising the adoption database to become more efficient; unitization of national adoption institutes in each province; selection of officials based on both factors of professional and complete mental health; alignment of the provisions and other related legal matters between the involved institutions; and utilization of media influence.

The third concept that emerged in this study was interactional solutions for family resilience. This concept is divided into five categories: cognitive-therapeutic interactions for mental health based on flexibility; raising the family's social capacity through active counseling; improving problem-solving skills; fostering family self-efficacy; and creating purposefulness and appropriate beliefs to predict a bright future.

Lastly, the fourth concept consisted of attitude-changing solutions, encompassing five categories: redefining social norms and facilitating the adoption of desired attitudes by employing social psychology principles; exploiting the power of media and influential figures; employing techniques derived from the psychology of learning (including social learning, sub-threshold conditioning, operant conditioning, and observational learning); establishing conducive conditions within the framework of individuals' cognitive dissonance to modify their attitudes (using principles from social psychology); and employing persuasion strategies.

Classification of experts' solutions for aiding families to adapt to their problems

As it was observed, four concepts concerning the adaptation of adoptive families to their problems emerged in the second stage of the research: explain the what, how, and why of adoption; operational solutions; interactional solutions for family resilience; and attitude-changing solutions.

Presentation of the model developed by the current research

According to the theory first presented by Strauss and Corbin [13], for a considerable amount of time, certain researchers advanced research according to six themes or axial codes, which included: casual conditions, context, central phenomenon, intervening conditions, strategies, and outcomes.

**Figure 1:**
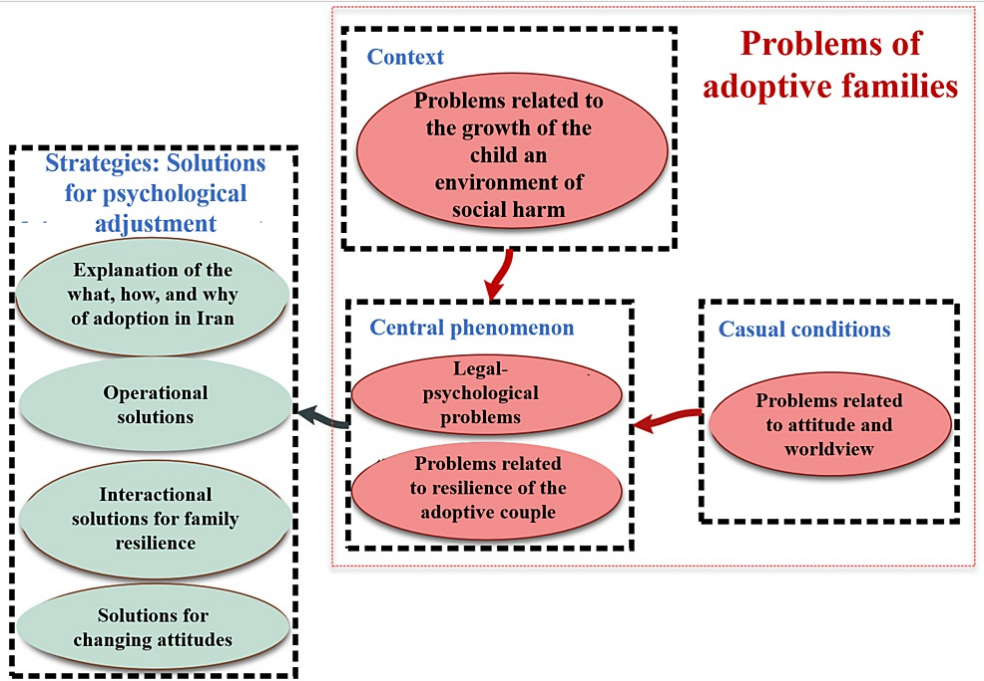
The study model derived from the grounded theory strategy The image has been created by the authors.

Nowadays, there is no need to adhere unreservedly to the old model, and researchers can present their systematic theory based on their understanding [19]. In the current research, by following the steps mentioned and attaining a deep understanding and intuition through a refined systematic process known as selective coding, the researchers have formulated a theory that has emerged from a grounded strategy of their own. This pattern is illustrated in Figure 1.

Validity and reliability of research

Eight approaches have been proposed to achieve the optimal level of validity for qualitative research, with two approaches assumed to be sufficient to warrant acceptable credibility for a research study. Moreover, several effective measures have been proposed to ensure the reliability of the research [20]. The strategies employed in this research to enhance validity were as follows: the researcher's sustained engagement with the research environment and ongoing observations within it; external oversight of the research through arbitration or third-party reporting; modification of hypotheses in tandem with the advancement of the inquiry in response to contradictory evidence; and the involvement of external evaluators at every stage. Therefore, even though two strategies could suffice to achieve adequate validity, this study employed four validity methods, which is indicative of appropriate validity. Additionally, with regard to measures aimed at enhancing reliability, the researcher employed a recorder during each interview and simultaneously made notes. The data were imported and analyzed by the qualitative analysis software MAXQDA 2020, with no data being overlooked. After coding was completed and the categories and concepts were extracted, the interviewers (families in the first phase and experts in the second phase) were consulted to ensure the accuracy of the initial coding process. The interviewees were also involved in re-checking the correctness of the coding process. Additionally, the collection process, as well as the analysis process, were regularly corrected and modified by an expert outside the circle of authors.

## Discussion

Four concepts were extracted in the problem identification step (first step. They were as follows: legal-psychological problems (weak coordination by and between organizations in charge, the need to appoint qualified and experienced experts, the absence of legal prohibitions against personalized and non-scientific opinions of experts, inadequate welfare training for adoptive parents, the lack of honesty and transparency required by organizations in charge regarding the essential part of the child's background, problems resulting from not recognizing single mothers as guardians, inequality between biological and non-biological parents regarding parental leave for the arrival of a child, and lack of adequate support for the parents of disabled children after being passed over to the family), the problems related to the growth of the child in an environment of social harm (biological-physiological issues, psychological issues, and educational issues), attitude and worldview (stereotypes, discriminations, prejudices, problems stemming from a lack of knowledge, and as a result, sustaining a flawed attitude), and the resilience of the adoptive couple (social competence, problem-solving, self-management, sense of purpose, and belief in a bright future).

Attitude and worldview issues, which were found to be causal conditions underlying other concepts, are in line with the findings of other studies [21].

The inconsistency of the results of this research and its innovation in providing the theoretical model may be attributed to the incorporation of a diverse sample group and experts from various fields such as psychology, law, philosophy, etc. This inclusion can be seen as a strength of grounded theory research.

Additionally, four concepts emerged from the second phase of the study, each containing proposals for further research. These were on the adaptation of adoptive families to their problems. In other words, from the second step, four groups of solutions for psychological adjustment were extracted: explanation of the what, how, and why related to adoption in Iran (clarification of concepts and demystification, research proposals to address other gaps in the knowledge infrastructure, and the necessity of critical thinking education), operational solutions (supervising the adoption database to become more efficient, unitization of adoption national institutes in each province, selection of officials based on both factors of professional and complete mental health, alignment of the provisions and other related legal matters between the involved institutions, and utilization of media influence are recommended), interactional solutions for family resilience (cognitive-therapeutic interactions for mental health based on flexibility, raising the family's social capacity through active counseling, improving problem-solving skills, fostering family self-efficacy, creating purposefulness, and appropriate beliefs to predict a bright future). Solutions for changing attitudes include redefining social norms and facilitating the adoption of desired attitudes; exploiting the power of media and influential figures; employing learning techniques; establishing conducive conditions within the framework of individuals' cognitive dissonance to modify their attitudes; and employing persuasion strategies.

It is recommended that media moguls pay special attention to adoption and prepare realistic programs under the supervision of psychologists and expert consultants in this field to foster a correct culture of adoption in society. Circumstances should be paved for volunteer adoptive parents to share their experiences and for researchers to conduct scientific research related to this field. This will facilitate making better decisions and ensuring that families have the right mental background to enter this path. Additionally, families' need for psychological support cannot be ignored. Legislators and law enforcers are advised to review the law, make any necessary amendments, and reevaluate the qualifications of those responsible for carrying out the law's executive duties. In addition, it is suggested that an adoption department be established in each provincial capital in the presence of efficient and specialized experts. If so, the continuous focus of the organization's legal and executive affairs can be conducted there in the presence of a designated trustee, thereby eliminating parallel work between organizations. As there is still a research gap in this area, it is recommended that researchers focus their attention on this group of parents and children in their studies. It is suggested that interdisciplinary research be conducted in light of this topic's expansive scope and multidisciplinary nature. Likewise, it is recommended that comparative studies of the legal status of adoption in Iran and other countries be conducted so that successful technical and expert experiences from other nations can be utilized. Once the pathology of the status quo in this field is identified, classified, and processed, the study's findings should be communicated to the decision-making, legislative, and executive centers as a measure for implementation.

## Conclusions

Considering the expanding recognition of adoption as a means to complete a family, it is evident that there is a need for research in this field. This research has taken an initial and significant step towards addressing adoption-related issues by identifying the problems adoptive families face and offering solutions to help them adapt to these challenges. Legislators and law enforcers, adoptive families, psychologists and consultants working in this field, and physicians specializing in infertility treatment can benefit from the findings of this study. Researchers and media moguls are recommended to give special attention to this issue to foster a culture of adoption and rectify attitudes surrounding it.
